# The effects of previous summer drought and fertilization on winter non-structural carbon reserves and spring leaf development of downy oak saplings

**DOI:** 10.3389/fpls.2022.1035191

**Published:** 2022-11-03

**Authors:** Xiaoyu Wang, Leonie Schönbeck, Arthur Gessler, Yue Yang, Andreas Rigling, Dapao Yu, Peng He, Maihe Li

**Affiliations:** ^1^ Jiyang College, Zhejiang Provincial Key Laboratory of Germplasm Innovation and Utilization for Garden Plants, Zhejiang Agriculture and Forestry University, Hangzhou, China; ^2^ Forest Dynamics, Swiss Federal Institute for Forest, Snow and Landscape Research, Birmensdorf, Switzerland; ^3^ Department of Botany and Plant Sciences, University of California, Riverside, Riverside, United States; ^4^ Plant Ecology Research Laboratory, School of Architecture, Civil and Environmental Engineering, Swiss Federal Institute of Technology Lausanne, Lausanne, Geneva, Switzerland; ^5^ Institute of Terrestrial Ecosystems, Eidgenössische Technische Hochschule Zürich (ETH Zürich), Zurich, Switzerland; ^6^ College of Ecology and Environment, Hainan University, Haikou, Hainan, China; ^7^ Institute of Applied Ecology, Chinese Academy of Sciences (CAS), Shenyang, Liaoning, China; ^8^ Tianjin Key Laboratory of Animal and Plant Resistance, College of Life Sciences, Tianjin Normal University, Tianjin, China; ^9^ Key Laboratory of Geographical Processes and Ecological Security in Changbai Mountains, School of Geographical Sciences, Northeast Normal University, Changchun, Jilin, China; ^10^ School of Life Science, Hebei University, Baoding, Hebei, China

**Keywords:** carbon consumption, carbon storage, leaf phenology, mobile carbohydrates, *Quercus pubescens*, overwinter, water deficit

## Abstract

It is still unknown whether the previous summer season drought and fertilization will affect the winter non-structural carbohydrate (NSC) reserves, spring leaf development, and mortality of trees in the next year. We, therefore, conducted an experiment with *Quercus pubescens* (downy oaks) saplings grown under four drought levels from field capacity (well-watered; ~25% volumetric water content) to wilting point (extreme drought; ~6%), in combination with two fertilizer treatments (0 vs. 50 kg/ha/year blended) for one growing season to answer this question. We measured the pre- and post-winter NSC, and calculated the over-winter NSC consumption in storage tissues (i.e. shoots and roots) following drought and fertilization treatment, and recorded the spring leaf phenology, leaf biomass, and mortality next year. The results showed that, irrespective of drought intensity, carbon reserves were abundant in storage tissues, especially in roots. Extreme drought did not significantly alter NSC levels in tissues, but delayed the spring leaf expansion and reduced the leaf biomass. Previous season fertilization promoted shoot NSC use in extreme drought-stressed saplings over winter (showing reduced carbon reserves in shoots after winter), but it also showed positive effects on survival next year. We conclude that: (1) drought-stressed downy oak saplings seem to be able to maintain sufficient mobile carbohydrates for survival, (2) fertilization can alleviate the negative effects of extreme drought on survival and recovery growth of tree saplings.

## Introduction

In the past decades, severe droughts and heatwaves have caused tree mortality and forest dieback across various biomes (Allen et al., 2010; Young et al., 2017), leading to profound changes in the structure and functioning of forest ecosystems on both global and local scale (Zhao and Running, 2010; Choat et al., 2018; Hartmann et al., 2018; Li et al., 2022). Natural drought events associated with increasing air temperature have been predicted to occur more frequently and to be of greater intensity in the future (IPCC, 2021), but it is still unclear whether and how trees tolerate and adapt to frequent droughts (Mcdowell et al., 2008; Allen et al., 2010; Mitchell et al., 2013; Adams et al., 2017; Choat et al., 2018).

Trees rely on stored non-structural carbohydrates (NSC, mainly composed of mobile sugars and starch) for growth and metabolisms (Hoch et al., 2003; Körner, 2003; Zhu et al., 2012a; Zhu et al., 2012b). Drought negatively affects photosynthesis and growth and can thus influence the levels of NSC in the growing season (Wiley, 2020). The NSC allocation patterns in response to drought have been extensively studied in trees during the growing season when both carbon supply and demand are directly affected (Li et al., 2008; Nardini et al., 2016; Li et al., 2018). However, to date, no consistent patterns in the NSC dynamics were found with some studies reporting decreased (Galiano et al., 2011; Woodruff et al., 2015) and others stable (Anderegg et al., 2012; Gruber et al., 2012; Schönbeck et al., 2018), or even higher (Piper, 2011; Piper and Fajardo, 2016; Kannenberg and Phillips, 2020) NSC concentrations in aboveground tissues during drought. The lack of unequivocal results might be attributed to varying tree size, species, and experiment time among different studies (Hoch, 2015). Contrary to aboveground parts, it is more commonly observed that root NSC decrease when trees are facing drought (Wiley, 2020). Li et al. (2018) analyzed 27 drought-related studies out of 57 NSC-related studies and reported that roots NSC decreased by 17.3%, but no NSC change was found in leaves and other aboveground woody tissues.

Drought may affect tissue NSC levels not only during the current growing season but also in the following winter and even next year (Lopez et al., 2007; Galvez et al., 2013). It was reported that previous growing-season drought can largely reduce the winter NSC reserves in roots of poplar seedlings (Galvez et al., 2013). In winter, NSC acts as an important substrate for sustaining respirational demands (Loescher et al., 1990), modifies cells membranes’ cryoprotective ability and improves cold hardiness by using sugars and sugar-derived compounds as osmoprotectants (Morin et al., 2007; Tixier et al., 2019). Therefore, low availability of pre-winter NSC will affect trees’ survival over winter, and low availability of post-winter NSC may strongly affect trees’ re-growth in early spring (Zhu et al., 2012b), especially for deciduous species (Lebon et al., 2008; Keller et al., 2010; Klein et al., 2016; Tixier et al., 2017). But even in evergreen species such as Scots pine, the winter NSC storage in fine roots and stem wood were found to be positively correlated with their shoot growth in the following season (Schönbeck et al., 2018). A warm 2015 winter in California caused a decline in winter NSC storage in trees, which finally induced growth decline of three conifer species in 2016 (Earles et al., 2018).

Nutrient availability in the soil might affect the fitness of trees under dry conditions. Previous studies provided conflicting results, showing that increased nutrient availability can either intensify or mitigate the negative effects of drought on trees (Kreuzwieser and Gessler, 2010; Gessler et al., 2017). Jacobs et al. (2004) reported that fertilization (blended fertilizer) impaired the root system development and drought resistance of drought-stressed Douglas-fir seedlings. However, Schönbeck et al. (2020) found that the negative effects of moderate drought intensity (but not severe drought) on Scots pine saplings could be compensated by increased nutrient availability (blended fertilizer, NPK) during drought. Zhang et al. (2021) reported that nitrogen addition increased net photosynthetic rate and stomatal conductance, but did not significantly change foliar NSC levels of drought-stressed *Quercus mongolica* and *Fraxinus mandshurica* saplings. To our knowledge, the effects of previous summer drought and fertilization on plant NSC over winter and spring leaf development next year have rarely been studied.

Therefore, we conducted a split-plot experiment with saplings of *Quercus pubescens* (downy oaks), a species has outstanding significance in most Mediterranean forest ecosystems (Kuster et al., 2013), grown under drought (four levels from well-watered to extreme drought with a volumetric water content of ~25%, 18%, 11% and 6%) and fertilizer (two levels: 0 vs. 50 kg/ha/year) treatments for one year (2016). We determined the NSC concentration in woody tissues in pre (Nov. 2016) and post-winter (March 2017), followed by an assessment of spring leaf development and leaf biomass of plants previously treated with drought and fertilization treatments. The study aimed to answer the questions of: (i) how the pre- and post-winter NSC reserves respond to drought events with different intensities (control, mild, strong, and extreme levels); (ii) whether the drought-stressed saplings can still mobilize and use the NSC storage in wood tissues during winter (by evaluating the over-winter NSC change); and (iii) whether and to what extent fertilization application affect winter NSC reserves, and how this change affects spring leaf development of previously drought-stressed oak saplings?

## Material and methods

### Experimental design and treatments

**Figure 1:**
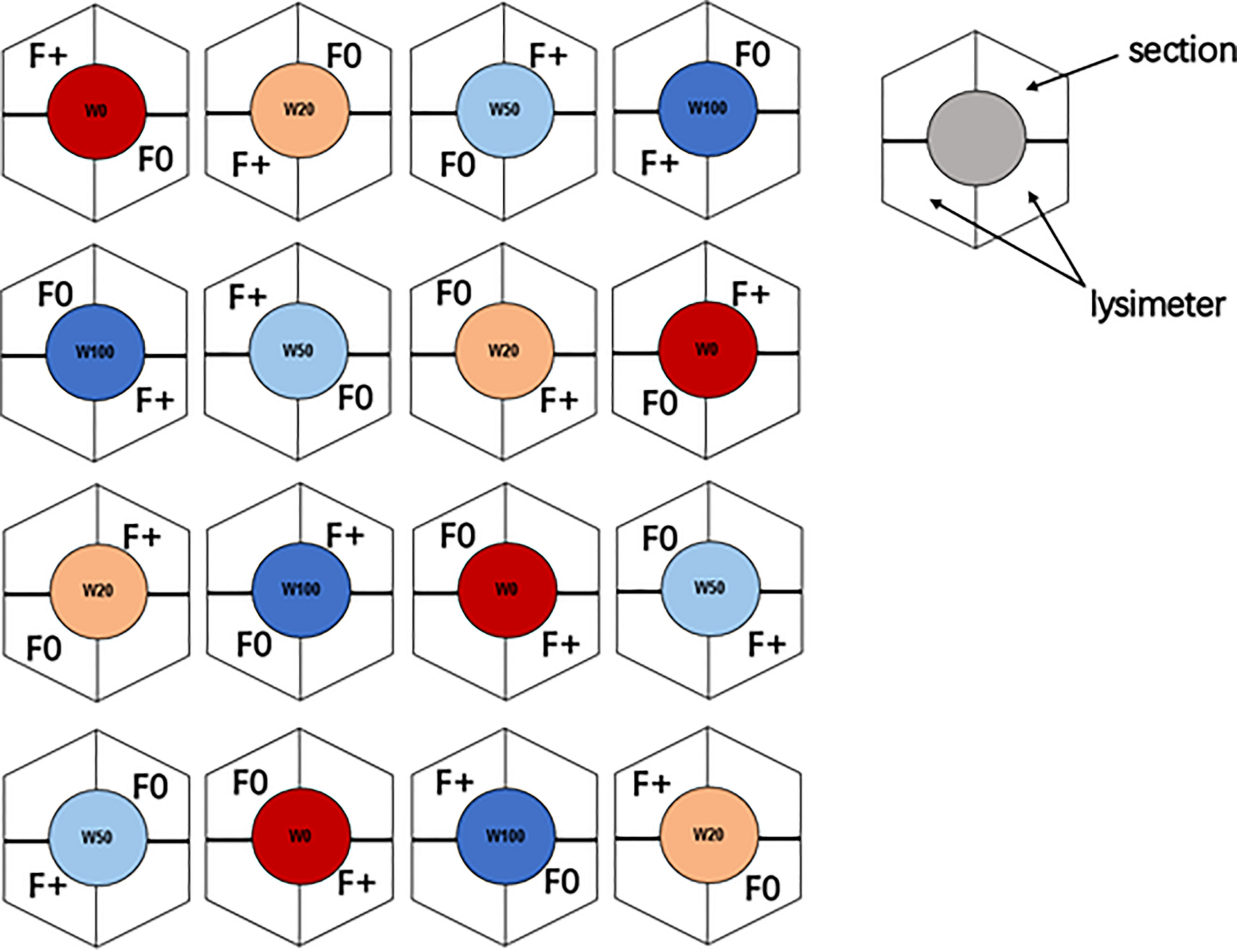
Experimental setup of the 16 open top chambers. Colors indicate the four different drought regimes: dark blue = W100 (close to field capacity), light blue = W50 (50% of field capacity), brown = W20 (20% of field capacity), and red = W0 (close to wilting point). Nutrient regime is represented with F0 (unfertilized) and F+ (fertilized) symbols.

The experiment was conducted in the model ecosystem facility (MODOEK) of the Swiss Federal Institute for Forest, Snow and Landscape Research WSL (47°21′48″ N, 8°27′23″ E, 545 m a.s.l.), Birmensdorf, Switzerland (**Figure 1**; **Figure S1**). The MODOEK unit consists of 16 hexagonal glass-walled open top chambers, each with 3 m in height and 6.0 m^2^ plantable area. Belowground, each chamber is divided into two lysimeters of 1.5 m depth and 3 m^2^ area, which were filled at the bottom with a 1 m layer of gravel for fast drainage and on top a 40 cm layer of calcareous sandy loam soil, divided by a mesh that is impermeable for roots but not for water (Kuster et al., 2013; Schönbeck et al., 2020). Each lysimeter was separated down to the mesh layer, with plexiglass into two equally sized sections (each has 1.5 m^2^ plantable area), two sections from different lysimeters were used for our experiment (**Figure 1**). Ten three-year-old *Quercus pubescens* Willd. (downy oak) saplings (20-25 cm in height, 0.3-0.5 cm in base diameter) were planted in each section in April, 2015, and they were grown with sufficient water supply in 2015.

The experiment was set up as split plot design considering both drought (4 levels) and fertilizer application (2 levels) treatments (**Figure 1**). From April 2016, each out of the 16 chambers was assigned one of the four water treatments (4 replicates for each water level): 100% (W100, corresponding to volumetric water content of ~25%), 50% (W50), 20% (W20), and 0% (W0, close to wilting point, volumetric water content of ~6%) of soil moisture at the field water capacity (Schönbeck et al., 2020). In April and July of 2016 and 2017, one section in each chamber was given 3 L water (F0: no fertilizer), the other one was fertilized with liquid fertilizer (F+: fertilizer treatment), close to 50 kg/ha/year (Wuxal, Universaldünger, NPK 4:4:3). The applied moisture was equal to 2 mm precipitation. The soil moisture and air temperature were automatically monitored at intervals of two hours (5 TM soil moisture and temperature logger, Metergroup, Munich, Germany) (data was shown in **Figure S2**).

### Pre-winter and post-winter harvest

We defined the average weekly soil temperatures at 5-cm depth below 5°C as non-growing season (winter) (**Figure S2**), the pre- and post-winter sampling works were conducted in November 2016, and March 2017, respectively. In each sampling season, trees from each chamber (both F0 and F+ section) were randomly selected and fully harvested, thus, 64 individuals (4 drought levels × 2 fertilization levels × 4 replicates × 2 seasons) were harvested (dug out from the soil). All harvested oak saplings were alive at the time of harvest. The saplings were then separated into aboveground tissues (shoots) and mixed roots (i.e., coarse and fine roots). All samples were dried to constant mass at 65°C for approximately 72 h and weighted thereafter. A subsample of the oven-dried material was ground to fine powder and stored at 4°C for further chemical analysis.

### Leaf phenology observation, mortality, and leaf biomass data collection

Spring leaf phenology was monitored in 2017, starting from April 3 (day 1) when bud swelling was first observed, to May 10 (day 37). Every two days within this period observations were carried out and three phenological stages were defined: (i) bud swelling, (ii) bud break, and (iii) leaf unfolding (Kuster et al., 2014). A stage was defined as reached for a given tree when > 50% of the buds showed the respective stage. For saplings that did not show any leaves (i.e. some saplings in the extreme dry W0 treatment) until May 10, 2017, a last observation was carried out in June 2017, to determine whether leaf phenology was delayed, or fully absent. If no leaves were present and the stem bark was brown and dry, the sapling was considered dead to calculate mortality. In July 2017 (mid-growing season), one sapling in each section was randomly selected for leaf biomass measurement.

### Chemical analysis

The NSC concentrations of all plant tissues were determined following the method of Wong (1990), modified as described in Hoch et al. (2002). NSC are defined as low molecular sugars and starch. About 10-12 mg of the dried plant material was boiled in 2 ml distilled water for 30 min. For determination of the total amount of NSC, 500 μl of the extract (including dissolved sugars and starch) were incubated with a fungal amyloglucosidase from *Aspergillus niger* (Sigma-Aldrich, St. Louis, MO, USA) for 15 h at 49°C to digest starch into glucose. For determination of soluble sugars, an aliquot of 200 μl was taken from the extract after centrifugation and treated with Invertase and Isomerase (Sigma-Aldrich, St. Louis, MO, USA) to degrade sucrose and convert fructose into glucose. The total amount of glucose in each sample (total NSC and soluble sugars) was determined photometrically at 340 nm in a 96-well microplate photometer (HR 7000, Hamilton, Reno, NE, USA) after enzymatic conversion to gluconate-6- phosphate (hexokinase reaction, hexokinase from Sigma Diagnostics, St. Louis, MO, USA). The concentration of starch was calculated as total NSC minus free sugars. Pure starch and glucose-, fructose- and sucrose- solutions (1 mg/ml) were used as standards and standard plant powder (Orchard leaves, Leco, St. Joseph, MI, USA) was included to control reproducibility of the extraction. NSC concentrations are expressed on a percent dry matter basis. P and K content in the tissues were also measured, i.e., 5-6 mg of ground plant material was weighted into tin capsules that were combusted in an Element analyzer (ICP-OES, Optima 7300 von Perkin-Elmer) for chemical analysis.

### Statistical analysis

The overwinter NSC changes were calculated as the difference between pre and post-winter NSC concentrations:


NSC change=PostNSC−PreNSC

Two-way ANOVA was then used to test how drought (W), fertilization (F), and their interaction (W × F) affect NSC concentrations in tissues at pre, post-winter, and the changes in NSC overwinter. Tukey HSD tests were carried out to examine pairwise differences when significant differences between drought or fertilization treatments were found, Bonferroni correction was used to adjust for multiple comparison.

For each oak individual (N = 252 in all chambers), the number of days required to reach the 3 different phenological states (Days) was calculated and a two-way ANOVA analysis (Days ~ Water * Fertilization) was used to test how drought and fertilization treatments affect the phenological development. To test the correlation between pre- and post-winter NSC (N = 32) on the one hand, and phenology (N=252) and biomass (N = 32) on the other, the number of days per phenological stage were averaged for each section. Linear regression models were established to study spring leaf development status and leaf biomass (July, 2017) in relation to NSC concentration in all tissues pre- and post-winter. Normality and homogeneity of standardized residuals were graphically checked on quantile-to-quantile and residual-vs-predicted plots. The significance level in this study was set at *p* < 0.05. All statistical tests and artworks were done with R software 4.1.2 (R Core Team, 2019, Vienna, Austria), under the RStudio environment.

## Results

### Pre-winter NSC concentrations

**Figure 2:**
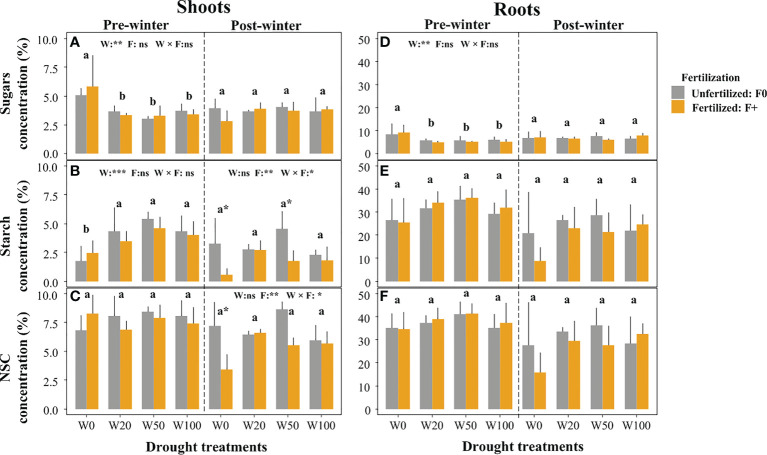
Effects of drought and fertilization on non-structural carbohydrates (NSC) in shoots and roots of downy oaks at pre winter (November, 2016) and post winter (March, 2017). **(A)** sugars in shoots, **(B)** starch in shoots, **(C)** the total NSC (sugars+starch) in shoots (left panel for pre-winter, and rightpanel for post-winter); **(D)** sugars in roots, **(E)** starch in roots, **(F)** the total NSC (sugars+starch) in roots. The drought treatments contain four levels: W100 (soil water capacity, ~25% VWC), W50, W20 and W0 (soil wilting point, ~6% VWC), W50 and W20 corresponding to approx. 50% and 20% field water capacity, respectively. Results of two-way ANOVA with drought (W) and fertilization (F) (their interaction effect was also considered) are given on the middle top of subfigures, using ns (non-significant), *p (< 0.05), ** (p < 0.01), or *** p( < 0.001) if significant effect was detected. Different lower-case letters represent significant differences in means among drought treatments tested with a Tukey *post-hoc* test, asterisks (on the bar) show significant differences between unfertilized (F0) and fertilized (F+) trees within the water treatment. Error bars indicate the SD of the mean values (*n* = 4). Note the y-axis scale change between shoots and roots.

Extreme drought (W0) resulted in significantly increased sugar levels in both shoots and roots (**Figures 2A, D**), and decreased starch concentration in shoots, compared to other drought treatments (**Figure 2B**, left panel). Pre-winter total NSC concentrations in roots and shoots were unaffected by drought (**Figures 2C, F**). There was no fertilization treatment (F) effect on pre-winter NSC measurements in both shoots and roots, although starch concentrations in shoots tended to be lower in fertilized saplings compared to unfertilized saplings except for the W0 treatment (**Figure 2B**).

### Post-winter NSC concentrations

Not drought but fertilization (F) treatments, and F interacting with drought significantly influenced shoots starch and the total NSC concentrations at post-winter (**Figures 2B, C**; right panel). The slight starch concentration differences between fertilization treatments observed pre-winter, were enlarged post-winter, mainly in W0 and W50. Here, fertilizer application led to a significant starch and total NSC (not significant for W50) reduction in the shoots (**Figures 2B, C**). Root NSC were unaffected by drought after winter, neither were shoot sugar concentrations (**Figures 2A, F**; **Table S1**).

### Over-winter NSC concentration changes

**Figure 3:**
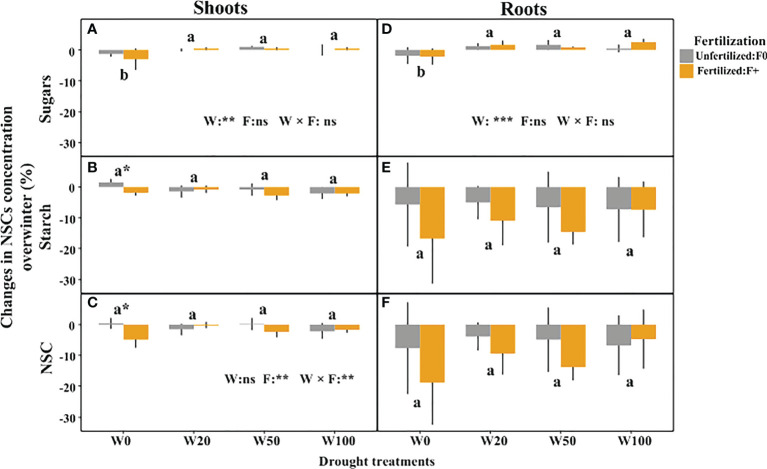
Changes in NSC concentrations overwinter in shoots and roots of downy oaks with different drought and fertilization treatments. **(A)** sugars changes in shoots, **(B)** starch changes in shoots, **(C)** the total NSC (sugars+starch) changes in shoots; **(D)** sugars changes in roots, **(E)** starch changes in roots, **(F)** thetotal NSC (sugars+starch) changes in roots. The drought treatments contain W100, W50, W20, and W0, represents 100%, 50%, 20%, and 0% of soil moisture at the field water capacity, respectively. Different lower cases letters represent significant differences in means among drought treatments tested by TukeyHSD, * signs show significant difference between unfertilized (F0) and fertilized (F+) trees within the water treatment. Error bars indicate the SD of the mean values (*n* = 4).

Shoots and roots tissues in all treatments consumed NSC over winter. The dynamics were an order of magnitude larger in the roots than in the shoots, where NSC concentrations in general were very low (**Figure 3**). Extreme drought (W0) caused a net over-winter reduction in sugar concentrations in both shoots and roots, while sugar levels increased in all other water treatments (**Figures 3A, D**; **Table S2**). A D × F interaction affected the changes in starch and NSC concentrations in shoots (**Figures 3B, C**; **Table S2**). This was caused by the slight increase of starch in the W0/F0 treatment, compared to a reduction in all other treatments (**Figure 3B**). Shoots of fertilized saplings in W0 consumed significant more NSC over winter, and fertilized saplings in W0, W20 and W50 treatments tended to (not significant) consume more root NSC reserves (**Figures 3C, E, F**).

### Spring phenology and leaf biomass

**Figure 4:**
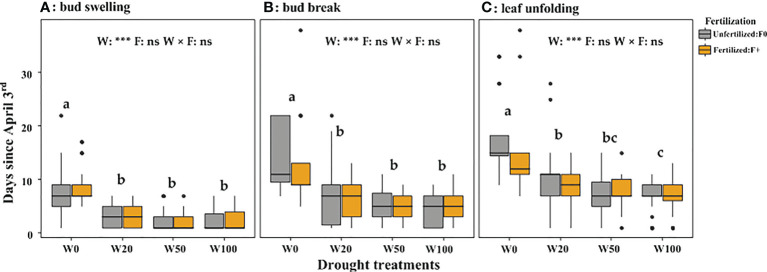
Number of days since April 3^rd^ 2017 until bud swelling **(A)**, bud break **(B)** or leaf unfolding **(C)** for trees in the different drought and fertilization treatments. The drought treatments depicted as W100, W50, W20, and W0, represents 100%, 50%, 20%, and 0% of soil moisture at the field water capacity, respectively. Different lower cases letters indicate statistical differences (*p* < 0.05) among drought treatments.

**Figure 5:**
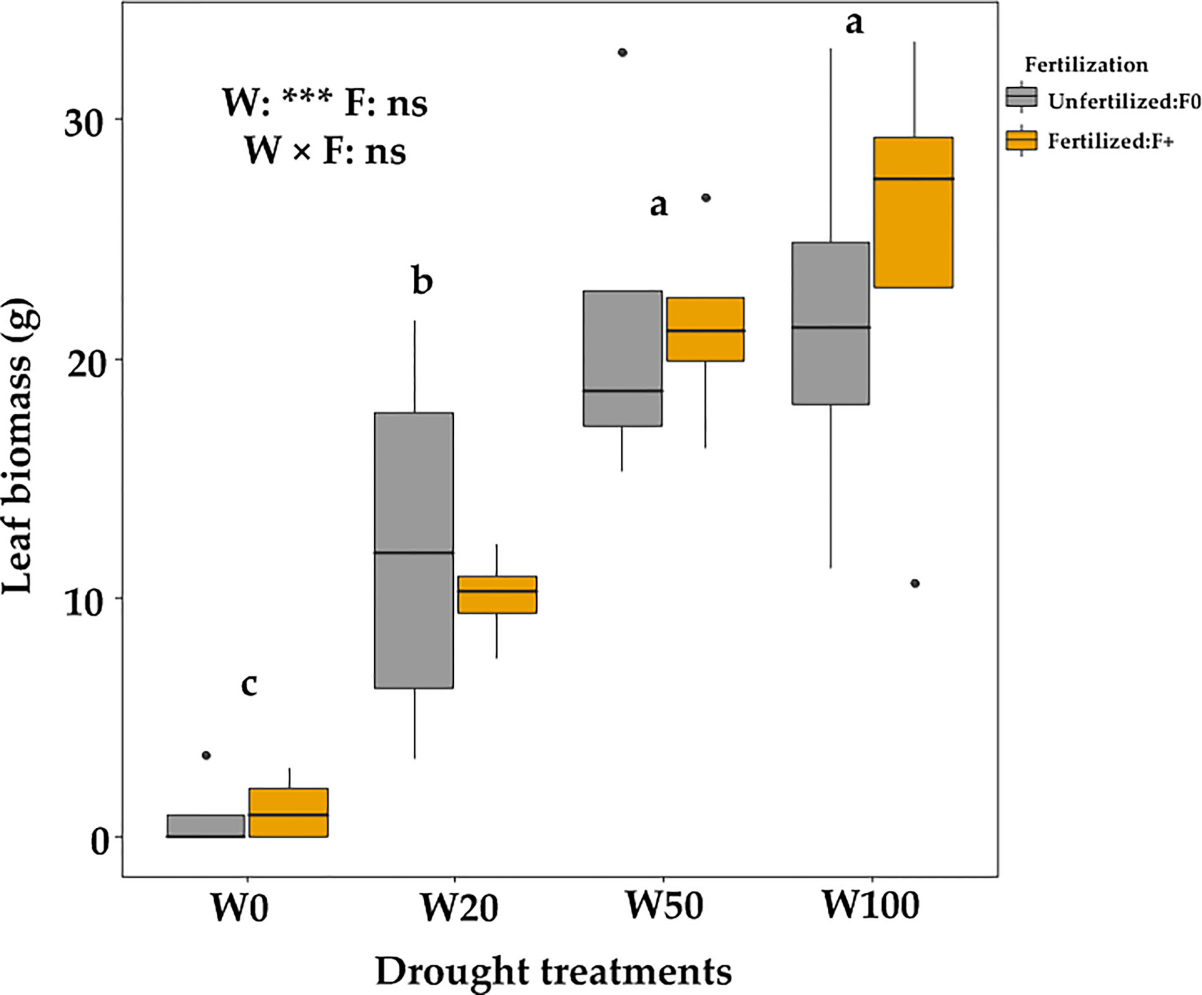
Leaf biomass of downy oaks (harvested in July, 2017) under different drought and fertilization treatments.

Extreme drought (W0) significantly delayed the spring leaf phenology (indicated by a higher number of days since April 3). Fertilizer application did not affect leaf phenology timing (**Figure 4**; **Table S3**) but increased the number of saplings that successfully expanded leaves in the W0 treatment (**Table 1**). The leaf biomass in W50 saplings did not significantly differ from W100 saplings, but W20 and W0 droughts significantly decreased the biomass of new leaves (**Figure 5**). Within each drought treatment, fertilization did not affect the leaf biomass in 2017 (**Figure 5**).

**Table 1:** Number of oaks which failed to reach the phenology stages of bud swelling, bud break and leaf unfolding, under mild (W50), strong (W20) and extreme (W0) drought treatments.

Saplings number	W0/F0	W0/F+	W20/F0	W20/F+	W50/F0	W50/F+
Total number	31	32	32	32	32	32
Fail in bud swelling	6 (19.4%)	7 (21.9%)	1 (3.1%)	0 (0%)	1 (3.1%)	0 (0%)
Fail in bud break	18 (58.1%)	14 (43.8%)	1 (3.1%)	0 (0%)	1 (3.1%)	0 (0%)
Fail in leaf unfolding	19 (61.3%)	16 (50.0%)	1 (3.1%)	0 (0%)	1 (3.1%)	0 (0%)

Data in the brackets represent to ratio of failure individuals. Only treatments where phenology failure was observed are shown, thus well-watered W100 data was not shown.

### Spring phenology and leaf growth in relation to pre- and post-winter NSC concentrations

The timing (days since April 3) of spring leaf phenology (bud swelling, bud break, leaf unfolding) and the leaf biomass in July were stronger correlated with pre-winter than with post-winter NSC concentrations (**Table 2**). Higher pre-winter starch concentrations in shoots were correlated with earlier commencement, while higher sugar concentrations in shoots and roots were correlated with a delay of all the leaf phenology stages. Higher starch concentrations in the roots, both pre- and post-winter, were only correlated with earlier bud swelling. Leaf biomass was positively related to pre-winter starch concentration in shoots and negatively related to pre-winter sugar concentrations in both shoots and roots (**Table 2**).

**Table 2:** Correlation between the number of days until different leaf development stages and leaf biomass in relation to non-structural carbohydrate (NSC) concentrations in shoots and roots at pre- and post-winter.

NSC indices	Parameters	Bud swelling	Bud break	Leaf unfolding	Leaf biomass
**Pre-winter**					
Shoots sugar	*Pr(>|t|)* *R^2^* *slope*	**< 0.001** **0.482** **-1.383**	**0.003** **0.266** **-1.569**	**0.004** **0.254** **-1.630**	**0.039** **0.134** **-2.951**
Shoots starch	*Pr(>|t|)* *R^2^* *slope*	**< 0.001** **0.343** **0.986**	**0.015** **0.188** **0.128**	**0.040** **0.138** **1.027**	**0.035** **0.139** **2.540**
Roots sugar	*Pr(>|t|)* *R^2^* *slope*	**0.005** **0.233** **-0.530**	**0.011** **0.205** **-0.748**	**0.035** **0.144** **-0.668**	**0.025** **0.157** **-1.761**
Roots starch	*Pr(>|t|)* *R^2^* *slope*	**0.013** **0.190** **0.164**	0.066 0.112	0.255 0.044	0.377 0.026
**Post-winter**					
Shoots sugar	*Pr(>|t|)* *R^2^* *slope*	0.055 0.117	0.206 0.054	**0.039** **0.139** **2.209**	0.480 0.017
Shoots starch	*Pr(>|t|)* *R^2^* *slope*	0.608 0.009	0.555 0.012	0.518 0.015	0.351 0.029
Roots sugar	*Pr(>|t|)* *R^2^* *slope*	0.486 0.016	0.592 0.010	0.939 < 0.001	0.305 0.035
Roots starch	*Pr(>|t|)* *R^2^* *slope*	**0.038** **0.136** **0.097**	0.289 0.039	0.223 0.051	0.665 0.006

-1 * DOY was used, thus positive and negative slope indicate a promotion or delay, respectively, of phenology with higher NSC. Slope of regression line is given if *Pr* < 0.05 (which is shown in bold).

## Discussion

### Impacts of previous drought and fertilization on pre-winter NSC

After one growing season, severely drought-stressed saplings did not show any signs of total NSC depletion, compared to the other drought and well-watered individuals (**Figure 2**). But they had strong reductions in leaf water content (**Figure S3**) and dry biomass in shoots and roots (**Table S4**, because of large variation in W100/F+ group, not statistically significant). Besides, it was observed that drought led to significantly decreased photosynthesis rate and leaf water potential in a comparable setting as well (Ouyang et al., 2021). Similar to our results, root NSC was not found to decrease during drought (Koppenaal et al., 1991; Zhang et al., 2015; Deng et al., 2019; Santos et al., 2021), while some others showed a drought-induced root NSC reduction (Li et al., 2018; Wiley, 2020). This suggests that downy oak can maintain stable NSC levels when exposing to strong drought (Wiley et al., 2013; Saffell et al., 2014), which is consistent with other studies on oak species (Nardini et al., 2016; Lavrič et al., 2017; Ouyang et al., 2021). Moreover, no fertilization effects on NSC were found across all drought treatments (**Figure 2**), which is inconsistent with results from a meta-analysis that nitrogen addition decreased NSC concentrations in roots (-5.0%), but increased NSC in aboveground wood (+6.1%) (Li et al., 2019). This difference might be related to the fertilizer used (blended fertilizer in the present study and nitrogen addition in that meta-analysis), and may also be related to the amount of fertilizer added (e.g. large variations in the meta-analysis).

The extreme drought treatment (W0) decreased starch concentrations and increased sugar concentrations but did not change the total NSC in shoots (and to a lesser extent in the roots). This suggests that drought stressed oaks converted starch into sugars for osmotic adjustment and potentially to repair xylem embolisms (Silva et al., 2010; Secchi and Zwieniecki, 2011). Moreover, Wong et al. (2009) indicated that the drought stressed trees rely more on osmoregulation (chemical changes) rather than physiological changes of cell properties in cold tolerance development for protecting cytoplasm macromolecules and membranes, serving as osmoregulatory function, and lowering freezing point, thus higher sugars were observed in W0 trees.

### Impacts of previous drought and fertilization on winter NSC consumption

In this study, drought treatments did not affect the overwinter consumption of the total NSC and starch in both shoots (**Figures 3C, B**) and roots (**Figures 3F, E**), but W0 significantly depleted sugars in both shoots and roots, whereas other drought treatments accumulated sugars in tissues (**Figures 3A, D**). Actually, a certain depletion level of NSC (especially starch) in winter is to be expected, because this is related to the maintenance of cellular respiration overwinter and to the starch-sugar conversion for frost protection (Bonhomme et al., 2005; Amico Roxas et al., 2021). In late winter, decline in sugar levels in wood tissues will support cellular respiration and metabolic activities to facilitate cellular changes with dehardening before budburst. For extreme drought stressed saplings, the conversion process of starch into sugars seemed to be impeded during winter (**Figure 3A**). This was also observed by Galvez et al. (2013), who showed that growing season drought hampered the conversion of starch into soluble sugars during winter for *Populus* saplings, and by Wong et al. (2009) who found that drought stressed sugar-maple trees declined sugar levels in branches, suggesting a lower degree of conversion of cellular components with hardening under drought stress.

Besides, a strong reduction in shoots’ NSC concentrations overwinter was observed in fertilized W0 (W0/F+) saplings, leading to a significantly lower post-winter NSC compared to individuals from all the other treatments. Compared to unfertilized saplings, there was comparable, generally decreased starch level in roots of fertilized trees in W0, W20 and W50 (also visible in W50 shoots). This result indicates that fertilization might promote starch use of drought stressed trees. Reduction of soil nutrient uptake due to drought will impair plant’s nutritional status and general functioning especially for osmotic regulation and membrane stability (Kreuzwieser and Gessler, 2010). Fertilizer application may mitigate nutrient shortage and improve the former physiological functions to a certain extent. In the present study, fertilization did not lead to higher N accumulation in both leaves sampled in September 2016 (**Table S5**) and July 2017, and in other wood tissues (Nardini et al., 2016; Ouyang et al., 2021). But fertilization significantly promoted K accumulation in shoots at pre-winter within each water treatment level (**Tables S6**, **S7**). As inorganic solute, K^+^ plays a central role in the water economy of plants, involves in osmotic regulation, maintenance of cell turgor and increases the stability of cell membranes (Saneoka et al., 2004; Ahanger et al., 2016). It also supports metabolic activity and frost hardiness by hydrolyzing starch into sugars (Elle and Sauter, 2000; Norozi et al., 2020). K has a favorable effect on water regulation and photosynthesis in plants (Snyder et al., 2005), but its effect on frost protection is unclear, as summarized by Charrier et al. (2015). In this study, the role and effects of K on key tree physiology functions including carbohydrate mobilization of drought stressed saplings were not specifically studied, which should be clarified in future studies.

### The phenology and its relation to pre and post-winter NSC

Extreme drought significantly delayed spring leaf phenology of downy oaks, which is in line with findings on other oak species (Vander Mijnsbrugge et al., 2016; Čehulić et al., 2019). Under the extreme drought in the present study, fertilization did not change the timing of spring leaf phenology but increased the survival rate of plants (**Table 3**, **Figure 4**), despite of very low post-winter shoot NSC concentrations in W0/F+ (~3% in W0/F+ vs. ~7% in W0/F0, *P* < 0.05). Previous studies proposed that hydraulic failure and carbon starvation are two possible physiological mechanisms for drought-induced tree mortality or growth decline (Mcdowell et al., 2008; Li et al., 2022), decreased NSC accompanied by decreased mortality with fertilization in the present study suggest that drought-induced mortality was driven by hydraulic failure, rather than carbon starvation, especially under high nutrient availability. But high nutrient availability might have promoted the mobilization and utilization of carbohydrates during winter (Wong et al., 2009; Galvez et al., 2013; Schönbeck et al., 2020). This led to lower post-winter NSC concentrations but help to repair cavitation and build new xylem to compensate cavitation induced by frost and extreme drought (Barbaroux and Breda, 2002), or promote sugars reverted back into the xylem sap and apoplastic free space, resulting in lower mortality. Therefore, fertilization reduced the saplings’ mortality rate of W0, recorded both in July 2017 (**Table 3**, 1.5 years drought, mortality rate: unfertilized 51.9% and fertilized 32.0%) and September 2018 (a follow up research with re-water treatment, mortality rate: unfertilized 78.6% and fertilized 51.2%) (Ouyang et al., 2021).

**Table 3:** Mortality rate of downy oaks saplings with different drought and fertilizer application treatments (investigated after 1.5 years drought, in June 2017).

Treatments	Number of dead	Total number	Mortality rate (%)
**W0/F0**	14	27	51.9
**W0/F+**	8	25	32.0
**W20/F0**	1	28	3.6
**W20/F+**	0	27	0
**W50/F0**	3	28	10.7
**W50/F+**	0	27	0
**W100/F0**	0	29	0
**W100/F+**	0	28	0

Pre-winter starch concentrations in shoots were positively correlated with the commencement of leaf phenology and with leaf biomass. Pre-winter sugars in shoots and roots were negatively correlated with the timings of all the stages and with leaf biomass (**Table 2**). Since pre-winter sugar and starch concentrations are highly related to the previous growing season water supply levels, we cannot confirm that pre-winter NSC affects leaf phenology and leaf growth directly or indirectly. A direct effect is supported by the results of Amico Roxas et al. (2021) who showed that through affecting bud endo- and ecto-dormancy periods (Fernandez et al., 2019; Tixier et al., 2019), fall NSC availability induces long-lasting effects on phenological timing of trees. However, we can also not exclude indirect effects: higher sugar levels (in shoots and roots) and lower starch (in the shoots) in the fall might indicate a still high demand for osmotic adjustment and potentially the need for cavitation refilling (Pérez-De-Lis et al., 2016; Savi et al., 2016), especially in W0. Bud swelling depends on sugar input from sapwood vessels, and xylem sap osmolarity also plays an important role in the generation of the stem pressure needed to reverse winter embolisms in early spring (Ameglio et al., 2001). A reduced ability to repair embolism in trees with low xylem sap sugars concentration could thus negatively affects the supply of water to bud swelling (Ameglio et al., 2001). Thus, stronger cavitation might lead to lower water supply of buds and thus delayed bud swelling and growth in the following spring.

Roots contribute largely to the total starch pool in downy oak (**Figure 2**), and might thus play an important role in the phenology process. Higher NSC (starch) in the roots might help to maintain root activity during winter to enhance water absorption in early spring. Moreover, root starch reserves might be later released and transported to the branches to support bud break (Tixier et al., 2017) and leaf formation in spring (Klein et al., 2016). Lower root starch reserves might thus delay these processes. The result shows that higher sugar concentrations are correlated with earlier leaf unfolding in the spring might be because shoot sugars are a direct carbon source for new leaves and can also support embolism repair (Pérez-De-Lis et al., 2016; Savi et al., 2016; Pagliarani et al., 2019; Tomasella et al., 2019), and thus increase the supply of water to swelling buds, which was also confirmed by Park et al. (2009) and Pérez-De-Lis et al. (2016).

## Conclusion

We examined previous summer drought and fertilization effects on carbon reserves over winter and spring leaf phenology of downy oak saplings next year. We found that, irrespective of drought intensity and the combined fertilization treatments, pre- and post-winter total NSC reserves were always abundant in storage tissues of previously treated saplings in expense of growth (Schönbeck et al., 2020; Ouyang et al., 2021). A mitigating effect of fertilization seemed to occur only when saplings grown under extreme drought, which is manifested in decreased mortality rate and increased recovery ability. This implies that increase in nutrient availability under extreme drought may alleviate phloem mobilization failure in some degree, and promote spring leaf development as a compensatory response. Based on this, we recommend to fertilize when plants subject to long-lasting extreme drought in temperate regions, to improve their drought resistance in the growing season, to increase the mobilization and utilization of sugar in winter, and to enhance the recovery ability next year.

## Data availability statement

The original contributions presented in the study are included in the article/**Supplementary Material**. Further inquiries can be directed to the corresponding authors.

## Author contributions

ML, LS, and AR designed, planned the experiment; XW, LS, and PH conducted the experiment and collected the data; XW, YY, DY and PH analyzed the data; ML led the manuscript writing; XW and ML wrote the first draft, XW, AG, LS, and ML revised the manuscript; ML finalized the article. All authors contributed to the article and approved the submitted version.

## Funding

This work was financially supported by a Swiss National Fund grant (31003A_157126/1, to ML), a Natural Science Foundation of Zhejiang Province (LQ21C030001, to XW) and National Natural Science Foundation of China (32001147, to PH). XW’s 12-month-research stay in Switzerland is financially supported by the China Scholarship Council, and PH’s 18-month-research stay in Switzerland is financially supported by Sino-Swiss Science and Technology Cooperation (SSSTC) program (EG06-032016) and the China Scholarship Council. Open access funding provided by WSL - Swiss Federal Institute For Forest, Snow And Landscape Research

## Acknowledgments

We sincerely appreciate suggestions for manuscript revisions from Deliang Lu, the editor and all the reviewers to improve quality of our paper.

## Conflict of interest

The authors declare that the research was conducted in the absence of any commercial or financial relationships that could be construed as a potential conflict of interest.

## Publisher’s note

All claims expressed in this article are solely those of the authors and do not necessarily represent those of their affiliated organizations, or those of the publisher, the editors and the reviewers. Any product that may be evaluated in this article, or claim that may be made by its manufacturer, is not guaranteed or endorsed by the publisher.
