# Supplementary material for: The effects of previous summer drought and fertilization on winter non-structural carbon reserves and spring leaf development of downy oak saplings
Source: Front Plant Sci. 2022 Nov 3;13:1035191. doi: 10.3389/fpls.2022.1035191 (PMC9669721; doi:10.3389/fpls.2022.1035191)
Supplement: Supplementary file 1 [file DataSheet_1.docx]

**Supplementary materials**

**Table S1. ANOVA results of models testing drought and fertilization treatment effects on non-structural carbohydrates (NSC) in shoots and roots of downy oaks harvested in November 2016 (pre winter), and March 2017 (post winter).** *F* and *p* values of two-way ANOVA analysis are given below.

|  | **Soluble sugars** | | **Starch** | | **NSC** | |  |
| --- | --- | --- | --- | --- | --- | --- | --- |
|  | *F* | *p* | *F* | *p* | *F* | *p* |  |
| **Pre-winter, November 2016** | | | | | | |  |
| **Shoots** | |  |  |  |  |  |  |
| Drought (W) | 7.310 | **0.001** | 7.898 | **<0.001** | 0.505 | 0.682 |  |
| Fertilize (F) | 0.075 | 0.786 | 0.576 | 0.455 | 0.245 | 0.625 |  |
| W × F | 0.443 | 0.724 | 0.714 | 0.553 | 1.580 | 0.220 |  |
| **Roots** |  |  |  |  |  |  |  |
| Drought (W) | 4.887 | **0.009** | 2.975 | 0.052 | 1.767 | 0.180 |  |
| Fertilize (F) | 0.294 | 0.593 | 0.250 | 0.622 | 0.139 | 0.712 |  |
| W × F | 0.195 | 0.898 | 0.145 | 0.932 | 0.083 | 0.969 |  |
| **Post-winter, March 2017** | | | | | | |  |
| **Shoots** | |  |  |  |  |  |  |
| Drought (W) | 0.838 | 0.486 | 2.102 | 0.126 | 2.336 | 0.099 |  |
| Fertilize (F) | 1.043 | 0.317 | 13.430 | **0.001** | 11.732 | **0.002** |  |
| W × F | 1.480 | 0.245 | 3.100 | **0.046** | 3.765 | **0.024** |  |
| **Roots** |  |  |  |  |  |  |  |
| Drought (W) | 0.184 | 0.906 | 2.172 | 0.118 | 1.955 | 0.148 |  |
| Fertilize (F) | 0.003 | 0.958 | 2.338 | 0.139 | 2.128 | 0.158 |  |
| W × F | 1.105 | 0.366 | 0.901 | 0.455 | 0.984 | 0.417 |  |

**Table S2. Two-way ANOVA table of drought and fertilization treatments affecting absolute change in non-structural carbohydrates (NSC) over winter.**

|  | **Soluble sugars** | | **Starch** | | **NSC** | |  |
| --- | --- | --- | --- | --- | --- | --- | --- |
|  | *F* | *p* | *F* | *p* | *F* | *p* |  |
| **Shoots** |  |  |  |  |  |  |  |
| Drought (W) | 6.334 | **0.003** | 2.881 | 0.057 | 0.930 | 0.441 |  |
| Fertilize (F) | 0.520 | 0.478 | 5.220 | **0.032** | 5.362 | **0.029** |  |
| W × F | 1.236 | 0.319 | 3.340 | **0.036** | 5.087 | **0.007** |  |
| **Roots** |  |  |  |  |  |  |  |
| Drought (W) | 9.085 | **<0.001** | 0.287 | 0.835 | 0.933 | 0.440 |  |
| Fertilize (F) | 0.452 | 0.508 | 3.046 | 0.094 | 2.832 | 0.105 |  |
| W × F | 1.334 | 0.287 | 0.416 | 0.743 | 0.693 | 0.566 |  |

**Table S3. Two-way ANOVA table of drought and fertilization treatments affecting the timing of three spring phenology stages and leaf biomass of trees (harvested on Jul, 2017).**

|  | **Drought(W)** |  | **Fertilize(F)** |  | **W×F** |  |
| --- | --- | --- | --- | --- | --- | --- |
|  | *F* | *p* | *F* | *p* | *F* | *p* |
| **Bud swelling** | **29.864** | **<0.001** | 0.042 | 0.840 | 0.015 | 0.997 |
| **Bud break** | **15.509** | **<0.001** | 0.001 | 0.982 | 0.108 | 0.954 |
| **Leaf unfolding** | **13.477** | **<0.001** | 0.466 | 0.502 | 0.475 | 0.703 |
| **Leaf biomass** | **20.152** | **<0.001** | 0.016 | 0.899 | 0.215 | 0.885 |

**Table S4. Dry biomass of trees (harvested on Nov, 2016) in the different drought and fertilization treatments.** Mean (SE) of biomass data is given below. The drought treatments depicted as W100, W50, W20, and W0, represents 100 %, 50 %, 20 %, and 0 % of soil moisture at the field water capacity, respectively. The fertilization treatments are indicated by unfertilized (F0) and fertilized (F+) levels.

| **Drought treatments** | **Fertilization treatments** | **N** | **Shoots**  **biomass (g)** | **Roots**  **biomass (g)** | **Total**  **biomass(g)** |
| --- | --- | --- | --- | --- | --- |
| W0 | F0 | 4 | 23.42 (3.42) | 29.45 (3.38) | 52.88 (6.76) |
|  | F+ | 4 | 22.58 (3.35) | 24.84 (3.76) | 47.42 (6.82) |
| W20 | F0 | 4 | 33.14 (8.77) | 68.51 (13.64) | 101.65 (22.35) |
|  | F+ | 4 | 32.14 (3.75) | 68.52 (6.70) | 100.65 (10.44) |
| W50 | F0 | 4 | 41.63 (3.47) | 65.48 (6.09) | 107.11 (9.01) |
|  | F+ | 4 | 34.89 (8.90) | 48.33 (4.71) | 83.22 (13.34) |
| W100 | F0 | 4 | 13.30 (0.86) | 32.78 (5.49) | 46.08 (5.38) |
|  | F+ | 4 | 72.66 (19.26) | 75.96 (11.41) | 148.62 (30.18) |

**Table S5. Two-way ANOVA table of drought and fertilization treatments affecting leaf nitrogen (N) content and leaf mass area (LMA) of oak sampled in Sep, 2016.**

|  | **Drought(W)** |  | **Fertilize(F)** |  | **W×F** |  |
| --- | --- | --- | --- | --- | --- | --- |
|  | *F* | *p* | *F* | *p* | *F* | *p* |
| Leaf N | 0.465 | 0.711 | 1.987 | 0.178 | 0.469 | 0.708 |
| LMA(g/m^2^) | 1.072 | 0.367 | 3.072 | 0.084 | 0.647 | 0.587 |

**Table S6. Two-way ANOVA table of drought and fertilization treatments affecting K and P content in shoots and roots of oak at pre and post winter.**

| **Season** | **Measurements** | **Drought(W)** |  | **Fertilize(F)** |  | **W×F** |  |
| --- | --- | --- | --- | --- | --- | --- | --- |
|  |  | *F* | *p* | *F* | *p* | *F* | *p* |
| Pre | Shoot K | 0.476 | 0.702 | **14.702** | **<0.001***** | 1.126 | 0.358 |
|  | Root K | 0.490 | 0.693 | 1.205 | 0.283 | 0.673 | 0.577 |
| Post | Shoot K | 0.183 | 0.907 | 1.429 | 0.244 | 0.992 | 0.413 |
|  | Root K | 0.296 | 0.828 | 3.082 | 0.092 | 0.684 | 0.571 |
| Pre | Shoot P | 2.265 | 0.107 | 0.354 | 0.557 | 0.464 | 0.710 |
|  | Root P | **5.079** | **0.007**** | 0.039 | 0.845 | 0.367 | 0.778 |
| Post | Shoot P | 1.292 | 0.300 | 0.558 | 0.462 | 1.142 | 0.352 |
|  | Root P | 0.535 | 0.663 | **6.003** | **0.022**** | 0.733 | 0.543 |

**Table S7. K content (mg/g) in shoots and roots of oak at pre and post winter.** The drought treatments depicted as W100, W50, W20, and W0, represents 100 %, 50 %, 20 %, and 0 % of soil moisture at the field water capacity, respectively. The nutrient treatments contain unfertilized (F0) and fertilized (F+) levels. Only pre-winter K content in the shoots was found to be significantly affected by nutrient treatment, TukeyHSD was then conducted to compare the difference among nutrient treatments, shown with case letters.

| **Drought** | **Fertilization** | **Pre winter** |  | **Post winter** |  |
| --- | --- | --- | --- | --- | --- |
|  |  | **Shoots** | **Roots** | **Shoots** | **Roots** |
| W0 | F0 | 3.307 (0.245)^b^ | 2.879 (0.189) | 3.938 (0.536) | 3.016 (0.254) |
|  | F+ | 4.494 (0.142)^a^ | 2.326 (0.115) | 3.790 (0.187) | 2.700 (0.050) |
| W20 | F0 | 3.295 (0.249)^b^ | 2.748 (0.157) | 3.571 (0.423) | 2.647 (0.277) |
|  | F+ | 3.543 (0.226)^a^ | 2.633 (0.094) | 4.935 (0.478) | 2.461 (0.063) |
| W50 | F0 | 2.806 (0.279)^b^ | 2.408 (0.331) | 3.983 (0.478) | 3.695 (0.462) |
|  | F+ | 4.380 (0.244)^a^ | 2.645 (0.136) | 3.587 (0.411) | 2.281 (0.149) |
| W100 | F0 | 2.942 (0.089)^b^ | 3.403 (0.301) | 2.853 (0.170) | 3.082 (0.247) |
|  | F+ | 4.596 (0.179)^a^ | 2.570 (0.175) | 4.636 (0.176) | 2.626 (0.112) |


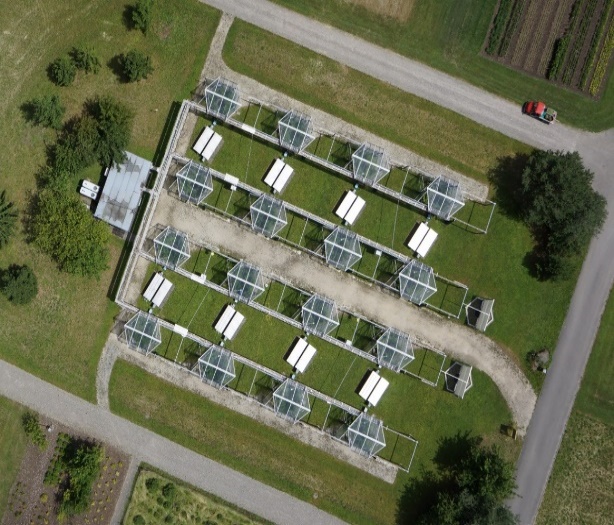

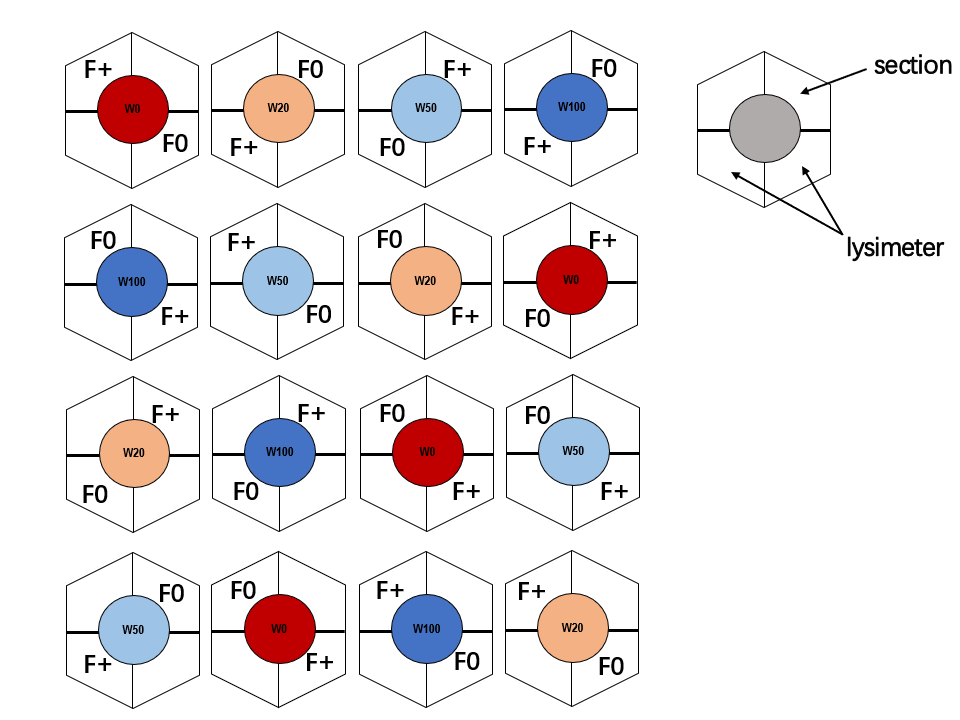


**A**


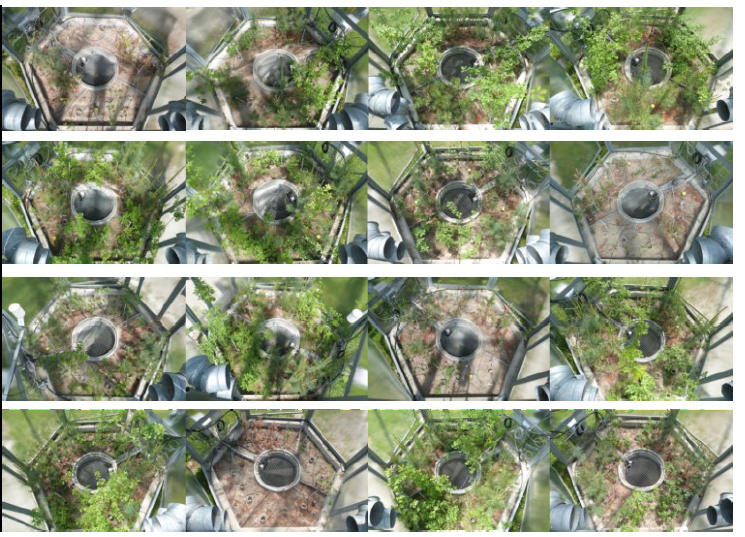


**B**


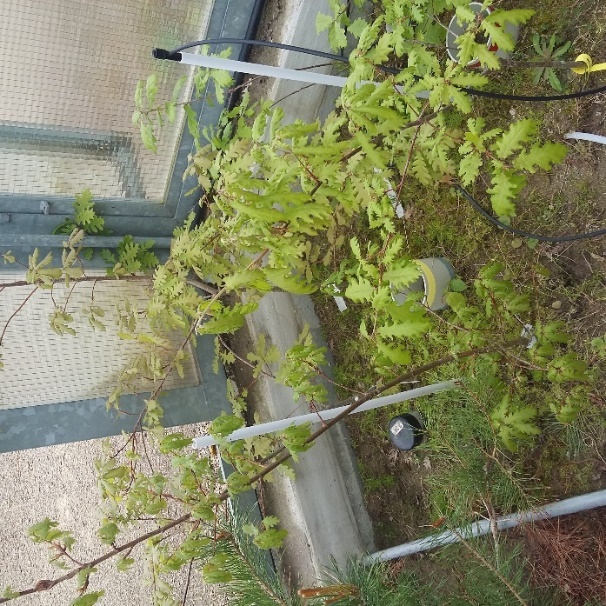

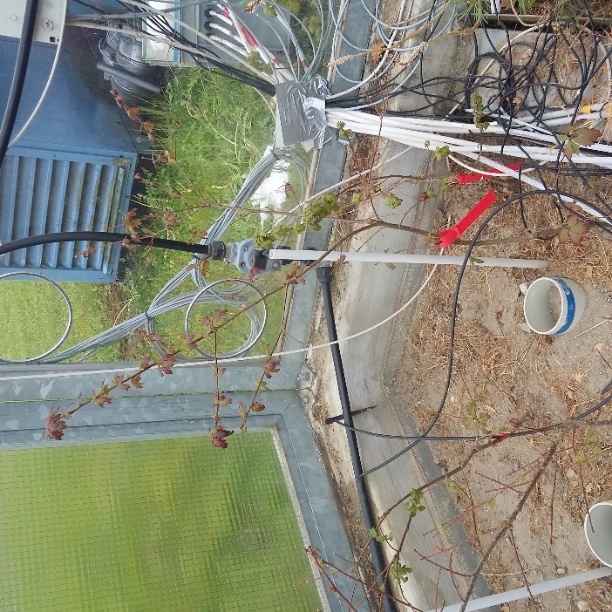


**C**

**Figure S1 Appearance of the Model Ecosystem Facility (MODOEK) program (A), photographs of plants in the 16 Open top chambers in May 2017, which is in the same order as indicated in the sketch map (B), mild (W50) and strong (W20) drought stressed downy oak saplings in April, 2017 (C).**

**Figure S2. The 5cm depth soil daily average temperature and soil relative water content (SWC, %) under different drought treatments.**

**Figure S3. Leaf water content (LWC, %) of downy oaks (sampled in Sep, 2016) with different drought and nutrient treatments.**

**Figure S4. Pre winter shoots K content (%) in relation to overwinter NSC decrease in the shoots for different drought-stressed downy oaks individuals.** Adjusted *R^2^*=0.172; *P* value: 0.011.
